# Comment on “Comparison of Electroacupuncture in Restrained and Unrestrained Rat Models”

**DOI:** 10.1155/2016/2157158

**Published:** 2016-01-06

**Authors:** Kang Hyun Yoon, Seung Min Kathy Lee, Jimin Park, Jungtae Leem, Jong Shin Woo, So Ra Lee, Kyung Hye Lee, Sanghoon Lee, Weon Kim

**Affiliations:** ^1^Department of Acupuncture and Moxibustion, College of Korean Medicine, Kyung Hee University, Seoul 02453, Republic of Korea; ^2^Department of Clinical Research of Korean Medicine, College of Korean Medicine, Kyung Hee University, Seoul 02453, Republic of Korea; ^3^Division of Cardiology, Department of Internal Medicine, Kyung Hee University Hospital, Kyung Hee University, Seoul 02447, Republic of Korea

Although acupuncture researchers have conducted studies using rats for decades, there is no established method for controlling the animals while performing acupuncture. Some choose to use general anesthesia [1]; some prefer light sedation [2]; many opt to use tools such as tubes, bags [3, 4], or clothing holders [5]; while others simply immobilize them by hand [6]. In 2013, a study by Zhang et al. [7] further compared the analgesic effects of electroacupuncture (EA) in rats using a novel nonrestraining method that was developed by one of the coauthors in 2011 [8]. Their results showed that the nonrestraining method had similar analgesic effects to the restraining model but reduced stress on rats while also giving researchers greater access to treatment intensities and acupuncture points. However, one acupuncture point that they did not explore was PC6, which is the most frequently stimulated treatment point in cardiovascular studies. Unlike other parts of the rat's body (outer part of the forearm, hind leg, or back), PC6 is located on the inner part of the forearm that frequently sweeps the ground, is open to a wide range of motion, and consists of thin muscle layers, resulting in difficulty with needle insertion and increased risk of bent or pulled-out needles. Furthermore, previous cardiovascular studies recommend performing EA on PC6 with a frequency of 2 Hz and verifying motor threshold response based on visible muscle twitches on the stimulated area [9–11], making the nonrestraining method even more challenging. Since we could not use the nonrestraining method and there were no studies exploring optimal experimental methods for cardiovascular rat models utilizing EA on PC6, we looked at the protective effects of EA in the restrained rat model and the anesthetized rat model of myocardial ischemia-reperfusion (IR) injury.

Thirty-two male Sprague-Dawley rats were randomly divided into an anesthetized (AN) group and a restrained (RS) group, which were further divided into four subgroups each (*n* = 4 for all subgroups) consisting of (1) the sham subgroup, which had their chest opened and the heart exposed but were sutured without further intervention; (2) the IR subgroup; (3) the IR + EA subgroup (EA on PC6, ST36 for 30 min); and (4) the IR + placebo subgroup (EA on nonacupoints without conduction of electricity). On the first day, IR injury was induced in all rats (except the sham subgroups) by placing a slipknot (5-0 silk) around the left anterior descending coronary artery and releasing it after 40 min of ischemia. During the whole process, rats were anesthetized with ketamine (75 mg/kg) and xylazine (2 mg/kg) and mechanically ventilated (Harvard Apparatus, MA, USA).

On the first day, the IR + EA subgroups in both AN and RS groups were anesthetized prior to surgery and preconditioned with EA on the left PC6 and ST36 starting 20 min after ischemia was induced to 10 min after the snare was loosened. The IR + placebo subgroups were treated with EA on nonacupoints without conduction of electricity. From the second day to the fifth day, the IR + EA subgroup in the AN group received EA for 30 min every day under anesthesia, with additional anesthesia administered intramuscularly if needed (but to a minimum degree); the IR + EA subgroup in the RN group received the same treatment immobilized in the restrainers. Similarly, placebo EA was conducted in the IR + placebo subgroups in both the AN and RS groups. All EA stimulation was administered with acupuncture needles (0.20 × 30 mm, Dongbang Acuneedle Co, Gyeonggi-Do, Korea) with a frequency of 2 Hz for 30 min.

To evaluate LV function, rats were anesthetized on the last day, and cardiac function was measured using two-dimensional transthoracic echocardiography (Vivid Q; GE Medical Systems, Milwaukee, WI, USA) with a 12 MHz probe. M-mode echocardiography of the LV was performed at the papillary muscle level, guided by two-dimensional short-axis images. LV cavity size was measured during at least three beats in each projection and averaged. The m-mode images yielded systolic and diastolic wall thicknesses (anterior and posterior), and LV end-systolic and end-diastolic diameters. LV fractional shortening was calculated as (LVEDD − LVESD)/LVEDD*∗*100.

All values are expressed as mean ± SE. Results were analyzed using the Kruskal-Wallis nonparametric test for multiple comparisons, and *p* values less than 0.05 were considered statistically significant.

In our findings, improvement in cardiac function was similar in the IR + EA subgroups of both AN and RS groups, but there were different results in the placebo subgroups. While the LVEF of the AN placebo subgroup did not improve (IR, 60.30 ± 5.49; IR + EA, 70.56 ± 4.42; IR + placebo, 54.88 ± 2.12, *p* < 0.05), the LVEF in the RS placebo subgroup improved so that the results did not differ significantly compared to the treatment group (IR, 58.83 ± 5.80; IR + EA, 67.03 ± 3.40; IR + placebo, 60.30 ± 5.49, *p* > 0.05).

Among many possible factors that could have produced the additional effects, we believe frequent reinsertion of acupuncture needles was a significant factor. In the restrained group, the rats frequently flexed their muscles to try to escape from the restrainers, which made the acupuncture needles fall out. Although the needles at ST36 mostly stayed intact, the ones at PC6 were a problem, as the needles were inserted to a shallower depth of approximately 5 mm. Every time the needles fell out, the researchers had to stick them back in, which increased stress and produced additional somatosensory stimulation. Unlike the RS placebo subgroup, the AN placebo subgroup (which did not require reinsertions) showed no significant improvement in LVEF, and in a confirmatory trial that limited the number of reinsertions per session to less than three, the LVEF of the RS placebo subgroup did not improve.

Our observation is noteworthy because in the study by Zhang et al. [7], the authors also report that they had to reinsert the needles several times when using the novel nonrestraining method. However, based on our findings, frequent reinsertion itself can contribute significantly to the final treatment effect and should be controlled regardless of how quickly and conveniently it can be performed.

Much more experiments to standardize and optimize research methods in acupuncture-related preclinical studies should be conducted. Novel methods to control rats must be continuously explored and researchers should know that differences in handling techniques can unwittingly expose well-designed studies to confounding factors, reproducibility failures, and limitations. Both of the chosen methods in our study do have their limitations, sedating animals prior to every acupuncture session is not feasible, and restraining rats also induces high levels of stress [12–14]. Using anesthesia for one or two days may be harmless, but results can be different when anesthetics are accumulated for several days. Furthermore, the difference between our study and the study by Zhang et al. [7] may be due to the different pathological rat models used and the different endpoints defined. For now, however, unless an improved nonrestraining method is developed that can be used for needling PC6, in acupuncture-related cardiovascular studies, researchers should try to minimize needle fall-outs, limit the number of reinsertions per session, or use general anesthesia if all factors cannot be adequately controlled.
